# College and Hospital Notes

**Published:** 1905-10

**Authors:** 


### COLLEGE AND HOSPITAL NOTES.

The Indiana Medical College has been made the school of medicine of Purdee University. The college was established in October, 1869, and for 35 years has given continuous instruction in medicine, during which time it has graduated more than 1,600 pupils. This union with Perdue University makes it a part of the State system of education.

---

The University of Buffalo opened the sixtieth year of its medical department Monday evening, September 26, 1905. .Dr. George F. Cott, clinical professor of otology, delivered the opening lectures to about 500 students, during the course of which he announced that the working day during the present college year would be one hour shorter, and that in consequence one month would be added to the collegiate course.

In the course of his address Dr. Cott advocated a college course of six years' length, stating his belief that the present four-year course was not long enough for thorough preparation. Dr. Cott said that the student should be given a year of hospital work before he was granted his degree.

